# Microcurrent-Mediated Modulation of Myofibroblasts for Cardiac Repair and Regeneration

**DOI:** 10.3390/ijms25063268

**Published:** 2024-03-13

**Authors:** Dipthi Bachamanda Somesh, Karsten Jürchott, Thomas Giesel, Thomas Töllner, Alexander Prehn, Jan-Peter Richters, Dragana Kosevic, Jesus Eduardo Rame, Peter Göttel, Johannes Müller

**Affiliations:** 1 Berlin Heals, 59-61 Knesebeck Str, 10719 Berlin, Germanytoellner@berlinheals.de (T.T.); richters@berlinheals.de (J.-P.R.);; 2 BIH Center for Regenerative Therapies, Regenerative Immunology and Aging, BIH Immunomics, Berlin Institute of Health at Charité, Universitätsmedizin Berlin, 13353 Berlin, Germany; karsten.juerchott@bih-charite.de; 3 Dedinje Cardiovascular Institute, Dedinje University Hospital, 11040 Belgrade, Serbia; 4 Jefferson Heart Institute, Thomas Jefferson University Hospital, Philadelphia, PA 19107, USA; eduardo.rame@jefferson.edu

**Keywords:** cardiovascular diseases, cardiac fibroblasts, fibrosis, myofibroblasts, microcurrent

## Abstract

Cardiovascular diseases are a significant cause of illness and death worldwide, often resulting in myofibroblast differentiation, pathological remodeling, and fibrosis, characterized by excessive extracellular matrix protein deposition. Treatment options for cardiac fibrosis that can effectively target myofibroblast activation and ECM deposition are limited, necessitating an unmet need for new therapeutic approaches. In recent years, microcurrent therapy has demonstrated promising therapeutic effects, showcasing its translational potential in cardiac care. This study therefore sought to investigate the effects of microcurrent therapy on cardiac myofibroblasts, aiming to unravel its potential as a treatment for cardiac fibrosis and heart failure. The experimental design involved the differentiation of primary rat cardiac fibroblasts into myofibroblasts. Subsequently, these cells were subjected to microcurrent (MC) treatment at 1 and 2 µA/cm^2^ DC with and without polarity reversal. We then investigated the impact of microcurrent treatment on myofibroblast cell behavior, including protein and gene expression, by performing various assays and analyses comparing them to untreated myofibroblasts and cardiac fibroblasts. The application of microcurrents resulted in distinct transcriptional signatures and improved cellular processes. Gene expression analysis showed alterations in myofibroblast markers, extracellular matrix components, and pro-inflammatory cytokines. These observations show signs of microcurrent-mediated reversal of myofibroblast phenotype, possibly reducing cardiac fibrosis, and providing insights for cardiac tissue repair.

## 1. Introduction

Heart failure is the ultimate fate of patients with cardiovascular diseases. Following heart injury, myocardial infarction, or chronic inflammation, deleterious remodeling of the heart wall ensues which leads to the formation of scar tissue predominantly comprised of myofibroblasts [1,2]. Adult cardiomyocytes (CMs) have little or no ability to regenerate further exacerbating this process [1,2]. Over time, myofibroblasts contribute to excessive collagen deposition, causing the heart to stiffen and leading to a progressive deterioration of heart function and ultimately heart failure [3,4,5]. Therefore, developing effective therapeutic strategies that target myofibroblasts and matrix proteins is of great importance. Current research endeavors aim to replace fibrous tissue with functional cells through regenerative approaches such as cellular therapy, tissue transplantation, biomaterials, and tissue engineering, with their clinical impact still only evolving [6,7,8].

The idea of bioelectricity and the significance of electric fields in biological systems are far from novel. It has been widely applied in various cells, tissue engineering, and regenerative medicine to provide electrical cues for tissue regeneration, cell proliferation, differentiation, or maturation [9,10,11]. In the context of heart failure treatment, electrical stimulation has been explored as a promising therapy to help strengthen and repair damaged heart tissue [12,13]. In vivo studies on rat hearts have shown that electric stimuli regulate the expression of ECM components and their inhibitors, accompanied by a significant decrease in pro-inflammatory cytokines [12,13]. Furthermore, recent studies with a Cardio-microcurrent device concluded that weak constant electrical current application to the heart is not only feasible and safe but also shows promising results in heart function and size [14,15]. Considering these promising outcomes observed with rat cardiomyocytes and preliminary clinical applications, this study specifically aimed to unveil the mechanism by which myofibroblasts respond to electrical stimulation to promote cardiac functional recovery.

Recognizing the central role of myofibroblasts in the cardiac wound-healing process, contributing to tissue remodeling and fibrosis, it is essential to understand the mechanisms and factors that regulate their phenotype, which could, in turn, provide essential insights for adapting their behavior across diverse clinical settings. Consequently, investigating the impact of microcurrent treatment on myofibroblasts is of paramount importance.

Therefore, this study sought to bridge this gap by investigating the effects of electrical microcurrents on cultured myofibroblast cells. By unraveling the intricate cellular and molecular mechanisms, this study aims to establish electrical stimulation as an innovative therapeutic approach for mitigating cardiac fibrosis and promoting tissue repair.

## 2. Results

### 2.1. Myofibroblasts Differentiate from Fibroblasts Efficiently Using a Combination of Low-Serum Medium and Passaging 

**Figure 1:**
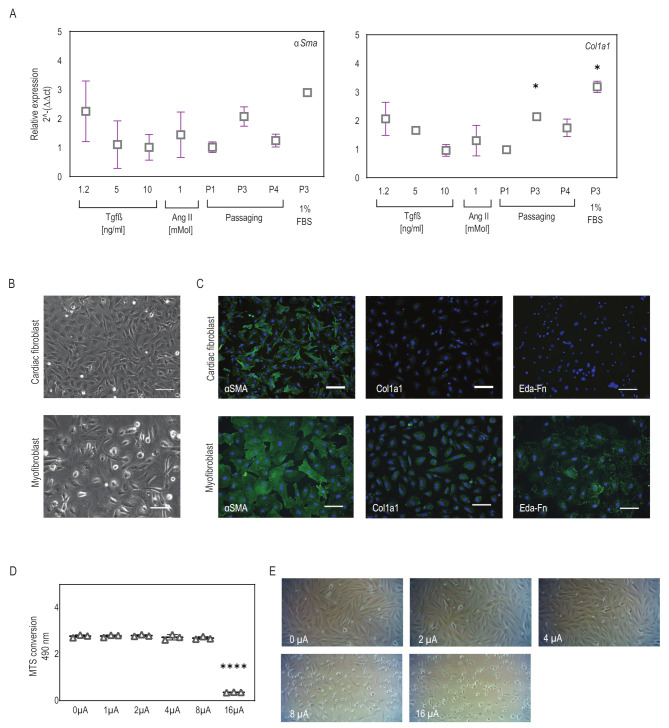
**Myofibroblasts differentiate and microcurrent treatment setup.** (**A**) Expression of myofibroblast markers *αSma* and *Col1a1* was observed in all conditions, indicating successful differentiation. Slightly higher expression was observed in the P3 + 1% FBS condition. Plot graphs present replicates and mean ± SD (*n* = 2–5). * *p* < 0.03. (**B**) Phase-contrast images reveal elongated and spread morphology upon differentiation to myofibroblasts. (**C**) Immunofluorescence staining for the myofibroblast markers αSma, Cola1a1, and Eda-Fn (green) comparing cardiac fibroblasts to differentiated myofibroblasts (P3 + 1% FBS condition). DAPI (blue) marks cell nuclei. Scale bars, 100 µm. (**D**,**E**) Cell viability assay for cardiac cells subjected to microcurrent application ranging from 0 to 16 µA/cm^2^ direct current. (**D**) The plot graph shows the effect of current densities on cell viability. Plot graphs present replicates and mean ± SD (*n* = 3). ns < 0.1, **** *p* < 0.0001. (**E**) The representative phase-contrast images display variations in cell density and instances of cell detachment and floating cells across the varying current densities.

Various previously established conditions were explored to investigate the most efficient differentiation strategy for fibroblasts into myofibroblasts. Differentiation efficiency was examined by analyzing the expression of myofibroblast markers such as anti-αSma and anti-Col1a1. qPCR analysis showed that *αSma* and *Col1a1* gene expression was observed in all the differentiation strategies tested, confirming the successful differentiation of CFs to myofibroblast-like cells (Figure 1A). Notably, the P3 + 1% FBS condition, where cells were continuously passaged up to P3 in low-serum medium, exhibited elevated αSma and Col1a1 expression compared to the other conditions (Figure 1A). Therefore, we determined that using a low-serum medium with passaging was an optimal condition for generating myofibroblast-like cells for future studies. This differentiation method was further validated through immunocytology for the markers *α*Sma, Col1a1, and Eda-Fn. We observed that these myofibroblasts displayed clear and bright Col1a1 and Eda-Fn marker expression compared to CFs (Figure 1C). In addition, the cell morphology of CFs changed from round epithelial patterns to elongated, fibrous cells, which was evident in both phase-contrast images and the immunofluorescence staining results (Figure 1B,C). After establishing an efficient in vitro culture system for differentiation, we sought to apply a microcurrent on these differentiated myofibroblasts and explore their effects.

### 2.2. Microcurrent Treatment

The effects of electrical stimulation on cellular proliferation and viability are critical considerations for applying microcurrent stimulation in heart repair. To determine the optimal current density, we applied current densities ranging from 0 to 16 μA/cm^2^ to CFs and monitored the resulting changes in cell viability and morphology. MTS assay showed that CFs remained highly viable after exposure to current densities of 0–8 μA/cm^2^ with no significant difference in viability (Figure 1D). Microscopic analysis revealed that at 0–4 μA/cm^2^ the current did not affect cell viability and showed confluent cell populations, whereas at 8 μA/cm^2^, fewer live cells and many floaters were observed, and at a current density of 16 μA/cm^2^ all the cells in the wells were dead (Figure 1E). Although differences in cell numbers and cell behavior were observed with currents ranging from 0 to 8 μA/cm^2^, these changes did not significantly impact cell viability as seen from the MTS assay. Therefore, for all subsequent experiments, 1 and 2 μA/cm^2^ microcurrents were utilized with and without a polarity reversal using a periodicity of 2000 s (1000 s positive polarity, 1000 s negative polarity).

### 2.3. Microcurrent Treatment Enhances Cell Viability, Restores Calcium Signaling, and Modulates Apoptosis

**Figure 2:**
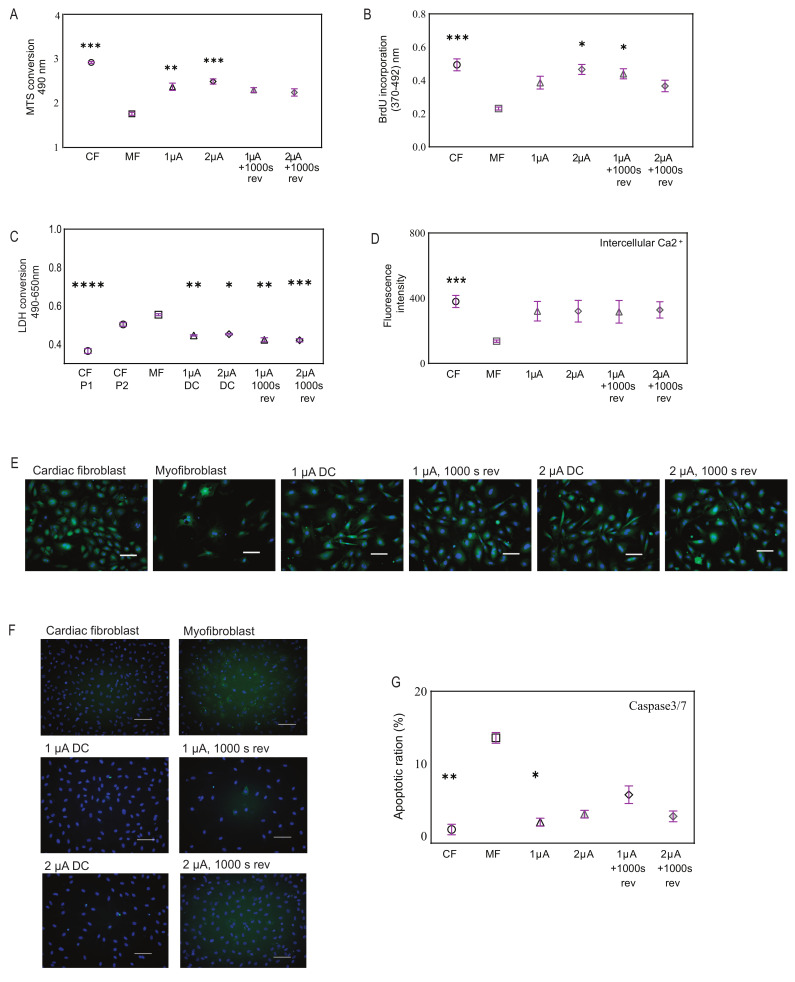
**Effects of microcurrent treatment on cellular processes.** (**A**) Cell viability for microcurrent-treated cells, comparing them to myofibroblasts and cardiac fibroblasts using MTS assay, (*n* = 6–12). (**B**) Cell growth was determined by measuring BrdU incorporation in the cell types. (**C**) Cellular cytotoxicity was assessed for microcurrent-treated cells, comparing them to myofibroblasts and cardiac fibroblasts using LHD assay, (*n* = 4–10). (**D**) The graph shows intercellular calcium signaling observed in all cell types but visibly low in untreated myofibroblasts. (**E**) Representative fluorescence images show intercellular calcium levels in cardiac fibroblasts, myofibroblasts, and microcurrent-treated cells (green) after staining the cells with Fluo-8 AM, a fluorescent labeling reagent. NucBlu (blue) marks cell nuclei. Scale bars, 100 µm. (**F**) Fluorescence images show apoptotic cells as determined by staining cardiac fibroblasts, myofibroblasts, and microcurrent-treated cells for Caspase3/7 (green). NucBlu (blue) marks cell nuclei. Scale bars, 100 µm. (**G**) The representative bar graph shows the percentage of apoptotic cells as determined by image cytometry, (*n* = 5). All Plot graphs represent replicates and mean ± SD. ns *<* 0.1, * *p* < 0.03, ** *p* < 0.002. *** *p* < 0.0002, **** *p* < 0.0001.

Next, we investigated the effect of electrical stimulation on myofibroblast behavior. Our results showed that the application of 1 and 2 μA/cm^2^ microcurrents with and without a polarity reversal to myofibroblasts demonstrated improvement in cell viability and was not toxic to the cells. Specifically, we observed that CFs after differentiation to myofibroblasts resulted in lower viability, and when treated with microcurrent, they showed an increase in cell metabolism, as observed with the MTS assay (Figure 2A). In line with the viability studies, the cell growth rate also showed improvement after microcurrent treatment as seen by BrdU incorporation (Figure 2B). In addition, the LDH assay revealed elevated LDH levels in both P2-CF and differentiated myofibroblasts, indicating high cytotoxicity. However, upon treatment of these myofibroblasts with microcurrent, a significant decrease in cellular cytotoxicity was observed irrespective of the microcurrent dose, as determined by a reduction in LDH release (Figure 2C).

Furthermore, our study investigated intracellular calcium expression in the different cell types via fluorescence intensity after cells were stained with Fluo-8, which increases significantly with rising intracellular calcium. The fluorescence intensities in CFs showed robust calcium signaling, while upon differentiation into myofibroblasts, there was a reduction in signaling. Notably, we observed restoration of calcium signaling in myofibroblasts after microcurrent treatment (Figure 2D). Microscopic observations also revealed similar outcomes (Figure 2E).

To further determine myofibroblast behavior before and after microcurrent treatment, we compared the expression levels of caspase 3/7, an essential protein involved in the execution phase of apoptosis. Microscopic observations revealed visible levels of caspase3/7+ (green) cells in myofibroblasts, which were reduced upon treatment with microcurrent. These were quantified to determine the ratio of apoptotic cells to total cells by hybrid cell count using a BZ-9000 fluorescence microscope and analyzer. Typically, in standard untreated cell cultures, 2–5% of cells have detectable levels of caspase-3 activity, and a similar percentage of apoptosis was observed in CFs microscopically. In contrast, untreated myofibroblasts showed a 13.5% higher level of caspase activity, indicating a significant increase in apoptosis compared to CFs. Microcurrent treatment of myofibroblasts led to a reduction in caspase activity (Figure 2F,G).

### 2.4. Microcurrent Treatment Alters the Myofibroblast Phenotype

**Figure 3:**
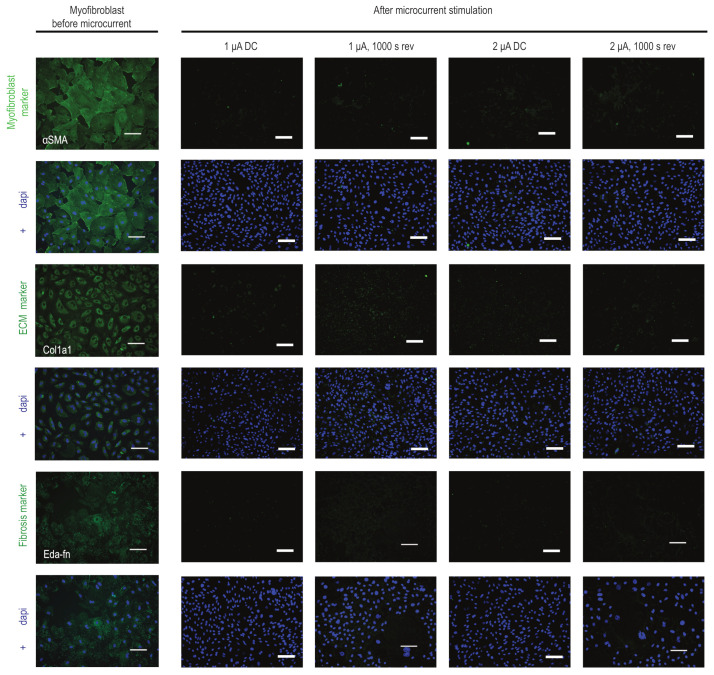
**Influence of microcurrent stimulation on myofibroblast phenotype.** Immunofluorescence staining for αSma, Cola1a1, and Eda-Fn (green) in myofibroblasts before and after microcurrent treatment with 1 μA/cm^2^ direct current (1 µA), 2 μA/cm^2^ direct current (2 µA), 1 μA/cm^2^ direct current + 1000 s polarity reversal (1 µA, 1000 s rev), and 2 μA/cm^2^ direct current + 1000 s polarity reversal (2 µA, 1000 s rev). After microcurrent treatment, a reduction in αSma-positive stress fibers and a reduction in the expression patterns of Cola1a1 and Eda-Fn markers in myofibroblasts were observed. DAPI (blue) marks cell nuclei. Scale bars, 100 µm. Colored images show a single channel for the markers (green) and merged images with DAPI (blue).

Further investigating the effect of microcurrent treatment on myofibroblasts, we examined the expression levels of a few markers in the microcurrent-treated cells. Our results reveal that the expression of αSma, a marker of myofibroblast activation, was visibly reduced in microcurrent-treated cells compared to untreated myofibroblasts and healthy CFs. Additionally, microcurrent treatment slightly reduced the expression levels of Col1a1 and Eda-Fn (Figure 3).

### 2.5. Transcriptome Analysis Reveals Unique Gene Expression Signatures and Cellular Responses in Microcurrent-Treated Cells

To expand our understanding of the effects of microcurrent treatment on myofibroblasts, we examined the global transcriptome of these cells using RNA sequencing and subsequent bioinformatics analysis (performed by Eurofins Genomics Europe Shared Services GmbH).

**Figure 4:**
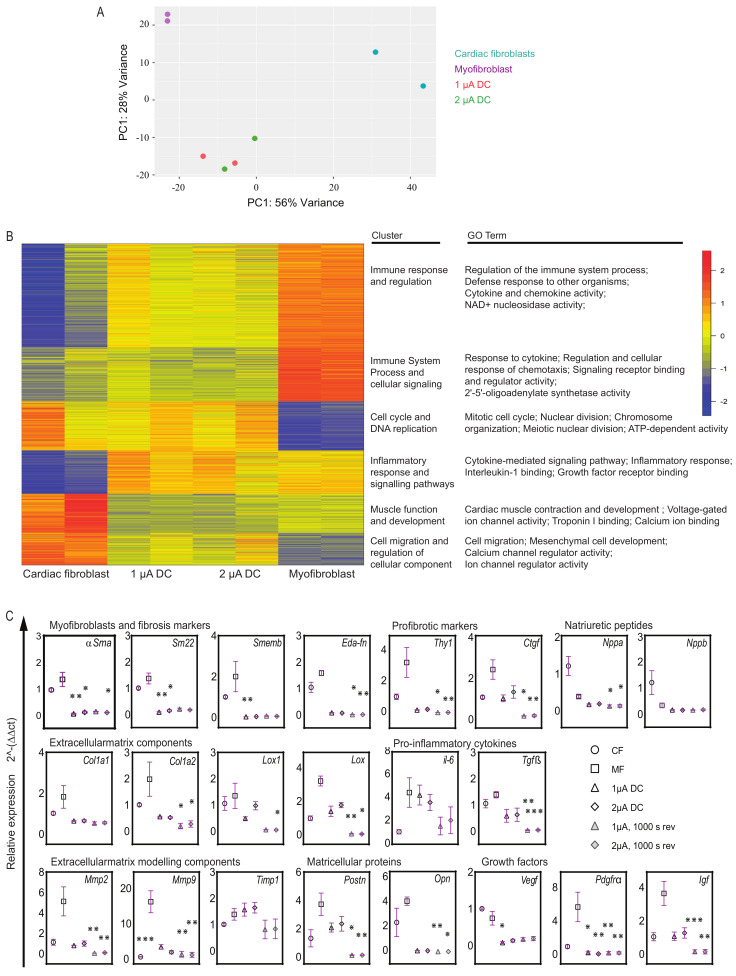
**Transcriptome analysis in microcurrent-treated cells.** (**A**) Principal component analysis of gene expression data generated by RNA sequencing from four sample groups: (1) healthy cardiac fibroblasts (CFs); (2) 1 μA/cm^2^ direct current microcurrent treated myofibroblasts (1 μA), (3) 2 μA/cm^2^ direct current microcurrent-treated myofibroblasts (2 μA), and (4) untreated myofibroblasts (MFs) (baseline control) (*n* = 2 for each sample group). The 2D principal component analysis plot presents the intra- and intergroup relationships by showing the first two principal components (PCs) that account for the majority of variation between samples. (**B**) K-means clustering and functional overrepresentation analysis of significant differentially expressed genes. The results are depicted as a heatmap with 6 clusters and a table listing their functional roles and gene ontology (GO) terms. They are selected from the top 25 terms with significant gene enrichment for each of the six k-means clusters. (**C**) Relative gene expression levels of myofibroblast markers, fibrosis markers, ECM structure and modeling components, natriuretic peptides, proinflammatory cytokines, matricellular proteins, and growth factors are shown for cardiac fibroblasts (CFs), untreated myofibroblasts (MFs) and microcurrent-treated myofibroblasts (1 μA direct current, 2 μA direct current, 1 μA direct current +1000 s polarity reversal and 2 μA direct current + 1000 s polarity reversal). Relative gene expression levels were quantified using the ∆ct method and normalized to three reference genes (*Gapdh* and *β-Act*). All plot graphs present replicates and mean ± SD (*n* = 3–6). ns *<* 0.1, * *p* < 0.03, ** *p* < 0.002, *** *p* < 0.001.

Principal component analysis (PCA) as a 2D graph shows the variations within and between sample groups. Our data revealed that the myofibroblasts treated with microcurrent displayed a distinct phenotype from untreated myofibroblasts as well as from CFs. The gene expression profiles of microcurrent-treated cells were unique and differed significantly from those of the myofibroblasts (Figure 4A). Next, to determine the functional roles of differentially expressed genes and the biological processes associated with them, we conducted k-means clustering, functional overrepresentation analysis, and gene ontology (GO) analysis. The results indicated that myofibroblasts exhibited upregulation of gene sets associated with immune responses, inflammation, defense mechanisms, and cellular signaling. However, microcurrent treatment was observed to reverse these alterations (Figure 4B). Furthermore, while myofibroblasts showed downregulation of cell cycle processes and DNA replication genes, microcurrent treatment was observed to upregulate these gene sets associated with cell cycle processes and DNA replication, comparable if not similar to the expression patterns observed in CFs. In addition, myofibroblasts showed a slight downregulation of the gene sets associated with muscle function compared to CFs. However, microcurrent treatment did not greatly alter muscle function genes but showed upregulation of cell migration and cellular process-regulation genes. The results revealed that microcurrent treatment leads to distinct transcriptional signatures in myofibroblasts, which differ from those observed in untreated myofibroblasts.

### 2.6. Microcurrent Treatment Modulates Gene Expression Profiles in Myofibroblasts

In light of the findings from RNA sequencing, and to obtain a more comprehensive understanding of gene expression profiles, qPCR was performed to validate and quantify the expression levels of specific genes of interest in myofibroblasts before and after microcurrent treatment. The expression patterns of a subset of genes revealed that applying a microcurrent, whether at 1 µA/cm^2^ or 2 µA/cm^2^ DC with or without polarity reversal to myofibroblasts, resulted in alterations in transcription levels compared to untreated myofibroblasts (Figure 4C). Microcurrent treatment led to a reduction in the expression levels of myofibroblast genes, such as *αSma*, *Sm22*, *and Smemb*, accompanied by a reduction in ECM structural components such as *Col1a1*, *Col1a2*, *Lox*, and *Lox1*. With regard to ECM modeling components, microcurrent treatment led to a marked reduction in *Mmp2* and *Mmp9* expression, and a diverse expression pattern of *Timp1* was observed. *Postn* and *Opn* key proteins known to regulate fibrosis were also downregulated after microcurrent treatment. Proinflammatory cytokine genes such as *Tgfß* and *il-6* were reduced following microcurrent treatment, while *Tnfα* showed little to no alterations. Furthermore, a marked decrease in *Igf* and *Pdgfrα* growth factors was observed.

## 3. Materials and Methods

### 3.1. Cell Culture

Primary neonatal cardiac cells and cardiac fibroblasts (CFs) from Wistar Kyoto rats were isolated as previously described [16,17], plated at 10,000 cells per cm^2^ onto dishes (CELLSTAR, Greiner Bio-One, Kremsmünster, Austria) and cultured in high-glucose Dulbecco’s modified eagle medium supplemented with 10% fetal bovine serum (FBS), 100 U/mL penicillin, 100 μg/mL streptomycin (Pen/Strep) (all Thermo Fisher Scientific, Waltham, MA, USA), and incubated at 37 °C and 5% CO_2_ in a humified incubator.

### 3.2. Cardiac Fibroblast to Myofibroblast Differentiation

Previously established differentiation conditions were tested to induce the differentiation of fibroblasts into myofibroblasts. Three different conditions were used: (i) fibroblasts were stimulated with transforming growth factor beta (TGF-β) and Angiotensin (ANG-II) as previously described [18,19]; (ii) fibroblasts were differentiated by passaging where confluent cells were split at a 1:3 ratio and maintained until passage 4 (P4) (P1, P3, and P4), and (iii) differentiation by passaging and starvation using low-serum medium. Confluent cells were split at a 1:3 ratio and maintained in continuous passage until P3 and the standard medium was replaced by a low-serum medium containing high-glucose Dulbecco’s modified eagle medium supplemented with 1% fetal bovine serum (FBS), 100 U/mL penicillin, and 100 μg/mL streptomycin (Pen/Strep). Differentiation was determined by qPCR analysis for genes alpha-smooth muscle actin (*αSma*), collagen type I alpha 1 (*Col1a1*). Cell morphology was assessed by microscopy and immunocytology for markers such as alpha-smooth muscle actin (anti-αSma antibody), collagen type I alpha 1 (anti-Col1a1 antibody), and fibronectin isoform ED-A (anti-Eda-Fn antibody).

### 3.3. Microcurrent Equipment and Treatment

The microcurrent equipment was designed with a specially adapted cover for a 24-well cell culture plate. The cover contains 24 Platinum/Iridium electrode pairs mounted on a circuit board (Supplementary Figure S1). Each electrode connects to a control unit with its dedicated constant current source for well configuration, allowing you to adjust electrical current in 0.1 µA/cm^2^ direct current (DC) increments and select polarity. To monitor the electrical values, the current and voltage of each well can be measured and logged.

To determine the optimal microcurrent dosage, cardiac fibroblasts were subjected to current dosages ranging from 0 to 16 µA/cm^2^ DC, and cell viability was assessed. Subsequently, to initiate microcurrent treatment, differentiated myofibroblast cells were plated at a density of 50,000 cells per well on gelatin-coated 24-well dishes (Corning CoStar, VWR). The following day, microcurrent was applied via the electrodes placed directly in the cell culture medium. Electric current was applied as 1 µA/cm^2^ DC, 2 µA/cm^2^ DC, 1 µA/cm^2^ DC with 1000 s polarity reversal, or 2 µA/cm^2^ DC with 1000 s polarity reversal for 72 h each. Medium change was performed every 24 h. In this study, cardiac fibroblasts (CFs) function as the control group, representing healthy cells, while myofibroblasts (MFs) serve as the baseline control, representing the diseased state.

### 3.4. Cell Behavior

(i). MTS assay

Cell viability was analyzed using the CellTiter 96 AQueous non-Radioactive Cell Proliferation Assay (Promega) a 3-(4,5-dimethylthiazol-2-yl)-5-(3-carboxymethoxyphenyl)-2-(4-sulfophenyl)-2H-tetrazolium (MTS) assay. Cells were incubated for 4 h according to the manufacturer’s instructions with tetrazolium compound MTS and an electron coupling reagent phenazine methosulfate (PMS), and the color reaction in the presence of viable cells was measured at 490 nm using the Anthos HT II plate reader (Anthos Mikrosysteme GmbH, Friesoythe, Lower Saxony, Germany).

(ii). BrdU Proliferation assay

Cell growth was analyzed using the BrdU Cell Proliferation ELISA Kit (Sigma Aldrich, St. Louis, MO, USA). To determine cell growth, cells were initially incubated with BrdU labeling reagent for 2 h followed by subsequent fixation and incubation with anti-BrdU antibody solution according to the manufacturer’s instructions. The absorbance of the samples was measured at 370 nm (reference wavelength 492 nm) using a SpectraMax Mini plate reader (Molecular Devices, San Jose, CA, USA).

(iii). LDH cytotoxicity assay

Determination of cellular cytotoxicity and cell necrosis was performed using the CyQUANT™ LDH Cytotoxicity Assay Kit (Thermo Fisher Scientific, Waltham, MA, USA). Supernatants from CFs, myofibroblasts, and microcurrent-treated cells were treated according to the manufacturer’s instructions and absorbance was measured at 490 nm and 680 nm using the Anthos HT II plate reader (Anthos Mikrosysteme GmbH, Lower Saxony). To determine LDH activity, the 680 nm absorbance value (background signal) was subtracted from the 490 nm absorbance value.

(iv). Calcium analysis

For the detection of intracellular calcium CFs, myofibroblasts and microcurrent-treated cells were stained with Fluo-8 AM (Abcam) and incubated at 37 °C for 30 min according to the manufacturer’s instructions. The fluorescence intensities were detected using a Tecan Infinite 200 Pro Fluorescence plate reader. Fluorescence images were obtained using a BZ-9000 fluorescence microscope (Keyence, Osaka, Japan).

(v). Caspase3/7 activity assay

Caspase 3/7 activity in cells was analyzed by CellEvent Caspase ReadyProbe (Invitrogen, Thermo Fisher Scientific, Waltham, MA, USA). Caspase reagent was added to the cells and incubated for 1 h according to the manufacturer’s instructions. Next, cells were stained with NucBlu Live ReadyProbes Reagent (Hoechst 33342) (Thermo Fisher Scientific, Waltham, MA, USA) to stain cell nuclei. Fluorescence images were acquired, and the ratio of apoptotic cells to total cells was determined by hybrid cell count using the BZ-9000 fluorescence microscope (Keyence, Osaka, Japan) and analyzer.

### 3.5. Immunocytology of Adherent Cells and Fluorescence Microscopy 

At indicated time points, cells were fixed with 4% PFA (paraformaldehyde) (Carl Roth) and incubated in permeabilization and blocking buffer (0.25% Triton X 100 and 5% BSA in Dulbecco’s phosphate-buffered saline). After that, cells were incubated with primary antibodies overnight at 4 °C and with secondary antibodies for 2 h at room temperature in the dark (Table 1). Finally, cells were stained with DAPI (Life Technologies, Thermo Fisher Scientific, Waltham, MA, USA) for nuclei staining. Fluorescence images were acquired using the BZ-9000 fluorescence microscope (Keyence). Differentiation was determined by markers such as alpha-smooth muscle actin (αSma), collagen type I alpha 1 (Col1a1), and fibronectin isoform ED-A (Eda-Fn).

### 3.6. RNA Isolation, RNA Sequencing, and Bioinformatics Analysis

Total RNA was extracted using an RNeasy mini kit (Qiagen, Hilden, Germany) and reverse-transcribed using the SuperScript III First-Strand Synthesis System (Invitrogen by Life Technologies/Thermo Fisher Scientific). Genomic DNA was removed with DNAse I (Sigma-Aldrich). RNA quantity and quality were assessed, and RNA sequencing was performed by Eurofins Genomics. The quality of raw data was controlled with FastQC v0.11.9. Reads from the fastq files were aligned to the rat transcriptome (rn6) using salmon (version 1.4.0) and the pre-calculated transcriptome index from refgenie (refgenomes.databio.org). All further data processing and analysis steps were completed in R (version 4.2.1). Raw counts were imported and summarized to counts per gene using tximeta (version 1.14.1). Data were normalized and further processed with DESeq2 (version 1.36.0). Variances were calculated for each gene across all samples, and the 1000 genes with the highest variance were used for a principal component analysis. Differentially expressed genes between two groups were determined after fitting models of negative binomial distributions to the count data. Raw *p*-values were adjusted for multiple testing using a false discovery rate (fdr). Genes with significant differential expression were selected by an adjusted *p*-value below 0.05 and a minimal absolute log_2_-fold change of one. Differentially expressed genes were subjected to K-means-clustering with 100 random start sets to avoid local minima, and over-representations of genes within these clusters in terms of the gene ontology system were determined with the topGO package (version 2.48.0) using the classical Fisher test with all annotated genes in the data set as background.

### 3.7. Reverse Transcription-Quantitative PCR 

For reverse transcription-quantitative PCR (RT-qPCR), reactions were prepared by combining cDNA samples (0.125 ng/µL reaction) with gene-specific primers (0.4 µM) and the PowerTrack SYBR Green Master Mix (Thermo Fisher Scientific). qPCR analyses were performed using the peqSTAR 96Q (Peqlab) with the following PCR program: initial denaturation (95 °C, 10 min), annealing, and extension (40 cycles: 95 °C, 15 s; 60 °C, 30 s; and 72 °C 30 s), with a melting curve program. Relative gene expression levels were determined utilizing the ΔΔCt method. ΔΔCt values were calculated by subtracting the ΔCt of each cell type from the ΔCt of cardiac fibroblasts (CFs), chosen as the reference sample for their representation of the healthy state. This approach facilitates a specific evaluation of microcurrent effects on myofibroblasts (MFs) within the cardiac fibrosis context, elucidating alterations in gene expression. *Gapdh* (Glyceraldehyde 3-phosphate dehydrogenase) and *β-Act* (beta-Actin) served as reference genes. Primers were synthesized by Eurofins Genomics (Table 2).

### 3.8. Statistical Analysis

Statistical analyses were performed using GraphPad Prism version 9.0 (GraphPad Software Inc., San Diego, CA, USA) employing a one-way ANOVA with the Kruskal-Wallis test, followed by Dunn’s multiple comparison tests to assess statistical significance among different treatment groups. In order to assess the impact of the microcurrent treatment, the mean of each microcurrent treatment group and cardiac fibroblasts were compared to the mean of MFs (myofibroblasts) in the baseline control group. All results are presented as mean ± standard deviation (SD). The number of replicates is given in the figure legends. *p*-values smaller than 0.05 were considered statistically significant. *p*-values for only significant results are shown.

## 4. Discussion 

In this study, we explored the therapeutic benefits of microcurrent treatment for heart injury. One of the main issues contributing to impaired heart function in the diseased heart is the loss of CMs and excessive myofibroblast formation [4]. Consequently, electrostimulating myofibroblasts emerges as a promising therapeutic approach as it targets a crucial cell type involved in the fibrotic response and impaired cardiac function during the wound-healing process. Given that cells naturally generate electrical signals as part of their physiological process, we believe microcurrent treatment can modulate this endogenous bioelectricity and enhance cellular functions to promote tissue repair and regeneration. Notably, to the best of our knowledge, this is the first report on the impact of a weak constant direct electrical current on the transcriptional regulation of cardiac myofibroblasts, expanding the current understanding of electrical stimulation in cardiology. To date, electrical stimulation studies on cardiology have focused on fibroblast cell migration in the context of wound healing or conductive biomaterials to promote CM maturation and reprogramming, or muscle contractions, overlooking its impact on myofibroblasts [20,21].

Our study of microcurrent treatment on cardiac myofibroblasts unveiled several interesting findings. Microcurrent stimulation demonstrated the ability to improve cell viability, reduce cellular cytotoxicity, and modulate apoptosis activation. These observations highlight the potential of microcurrent stimulation to promote a favorable cellular response. Excessive myofibroblast activation, often associated with cardiac fibrosis, detrimentally affects adjacent cells and tissues, potentially triggering cell death and increased cytotoxicity [22]. Furthermore, research indicates the involvement of diverse cell types in fibrotic progression, revealing a complex network of cellular interactions and highlighting a high degree of interconnectedness [23]. Investigating the impact of myofibroblasts on these parameters is, therefore, crucial for understanding their intricate role in cardiac health and disease. Additionally, calcium signaling, known for its vital role in heart health and pathological conditions, introduces another layer of complexity [24]. Although our study acknowledged the influence of microcurrent treatment on calcium signaling, suggesting a restorative effect of calcium signaling in myofibroblasts, it did not make significant progress in unraveling its complexities due to the involvement of numerous receptors, transporters, and ion channels. Nevertheless, the observed positive impact on calcium signaling indicates a promising avenue for further exploration.

Building on these promising results, our exploration was extended to uncover additional insights into the molecular mechanisms influenced by microcurrent treatment on cardiac myofibroblasts. Microcurrent treatment demonstrated a remarkable ability to effectively prevent myofibroblast differentiation, reduce collagen marker expression, and suppress the stabilization of the myofibroblast phenotype. These processes reveal the therapeutic potential of microcurrent treatment to disrupt the fibrotic cascade and attenuate the pathological processes associated with excessive collagen deposition in cardiac tissue. Excessive ECM deposition in cardiac tissue is a characteristic feature of cardiac fibrosis and myofibroblast phenotype [25]. Interestingly, a decrease in collagen marker expression and additional myofibroblast markers and genes such as *aSma*, *Sm22*, and *Smemb* [26,27,28] in myofibroblasts after microcurrent treatment was observed. Notably, consistent with previous findings [29], the expression of *Mmp’s* was low in CFs but elevated in myofibroblasts during the process of remodeling; however, microcurrent treatment resulted in a reduction in *Mmp* expression and activity. It is also worth noting that the expression of *Tgf-β*, a key player in fibroblast differentiation to myofibroblasts, collagen regulation, and the development of fibrosis [5,30,31,32], was decreased after microcurrent treatment. This aligns with previous research on spontaneously hypertensive rats (SHRs) [13], indicating a potential mechanism through which microcurrent therapy prevents myofibroblast differentiation, modulates behavior, and reduces inflammation and fibrosis. Several studies demonstrate the downregulation of α-*Sma* in cultured differentiated myofibroblasts in response to a variety of factors, like TGFβ1 antagonists, growth factors, and matrix compliance [33]. While the reversal of myofibroblasts has not been previously documented, our investigation introduces a novel aspect by demonstrating that microcurrent treatment not only achieves downregulation of α-Sma but also has the potential for the reversal of myofibroblasts, highlighting the transformative effects of this therapy on cardiac fibrosis.

Electrical stimulation can have diverse effects on cells, such as facilitating myofibroblast differentiation for wound healing [34,35,36] or increasing cell proliferation and altering ECM components [20,36,37]. The different outcomes can be attributed to several factors, such as differences in the cell types studied and the current form. Our research specifically examined differentiated myofibroblasts, as opposed to the more commonly studied fibroblasts. Furthermore, significant gene expression variations occurred with polarity reversal during the experiment, highlighting the impact of electric current type and intensity on gene modulation. Several clinical studies on microcurrents over the years have shown that applying polarity reversal to various wounds led to accelerated healing or regeneration [38,39]. However, further investigation is needed to unravel the complex interplay between electric current and the molecular pathways that regulate myofibroblasts.

Further investigation into the global transcriptome analysis highlights the distinct gene expression profiles and functional roles, suggesting that microcurrent treatment can influence myofibroblast behavior. This modulation involves the downregulation of immune responses, inflammation, and defense mechanisms indicating an anti-inflammatory effect, while promoting cell cycle processes and morphogenesis, indicating that microcurrent treatment may influence tissue repair. It is well-established that in healing infarcts, suppressing pro-fibrotic pathways is crucial for preventing uncontrolled fibrosis [5]. Notably, microcurrent treatment was observed to reverse these alterations associated with the active phase of tissue healing that contributes to fibrosis, suggesting anti-fibrotic and anti-inflammatory effects. Furthermore, acknowledging the heterogeneity of the myofibroblast population at different stages of wound healing provides insight into the gene expression profiles of specific gene sets. The downregulation of gene sets related to cell cycle processes and DNA replication genes can be attributed to the characteristics of fully differentiated myofibroblasts. These cells are typically in a contractile and differentiated state rather than actively proliferating [40].

Considering the broader context, our exploration into the effects of microcurrent therapy on cardiac myofibroblasts aligns with recent discoveries in a 3D-engineered cardiac tissue model investigating the impact of Cardiac Contractility Modulation (CCM) on heart function, wherein non-excitatory electrical stimulations were applied [41]. Microcurrent therapy shows promise in disrupting the fibrotic cascade linked to excessive collagen deposition in cardiac tissue as observed in our findings. Similarly, the CCM study demonstrates potential, unveiling stronger contractions, heightened calcium activity mirroring our observations, and influencing the expression of genes associated with heart failure. Together, these findings contribute to a more comprehensive understanding of electrical stimulation therapies for heart-related conditions.

Furthermore, it is important to note that devices for electrical stimulation of organs with a weak constant electrical current are in clinical testing and have shown promising results [14,15]. There is, however, a dire need to understand the underlying mechanisms of how cells behave upon electrical stimulation. We believe our study, combined with these existing human studies [14,15], contributes significantly to the understanding of microcurrent therapy for cardiac care. Additionally, findings from prior studies with CMs [12,13] along with our current study demonstrate that microcurrent treatment not only exerts a positive influence on the gene expression patterns of myofibroblasts but also promotes CM proliferation. These collective findings emphasize the transformative and regenerative potential of microcurrent treatment in addressing cardiac conditions, eliminating the need for cell transplantation or gene delivery. Nevertheless, one must be aware of the limited understanding of the physiological and cellular network. Although the heart consists of several cell types, most in vitro studies (including ours) focus solely on individual, isolated cell types and fail to reveal the physiological interaction between the different cells. A deeper understanding of these interactions and their contributions to the healing process is critical for developing new strategies to treat heart diseases and injuries.

## 5. Conclusions

Microcurrent stimulation shows promise for cardiac therapy, offering profound insights into its capacity to actively modulate cardiac fibrosis and promote tissue regeneration. Microcurrent treatment demonstrated the ability to reduce collagen deposition, suppress the stabilization of the myofibroblast phenotype, and even reverse myofibroblasts, eliciting anti-fibrotic and anti-inflammatory effects. Furthermore, it resulted in distinct transcriptional signatures and improved cellular processes like cell viability and intercellular calcium signaling. These promising outcomes have been supported by encouraging preliminary clinical applications, further highlighting the translational potential of microcurrent therapy in advancing cardiac care. Our study serves as a pivotal milestone, paving the way for future research.

## Figures and Tables

**Table 1:** Immunofluorescence staining antibody details.

Immunogen, Conjugate	Host	Dilution Factor	Product Details
Primary antibodies			
α-SMA (Anti-α smooth muscle)	Mouse	1:200	A2547, Sigma Aldrich
Col1a1 (Anti-Collagen Type I)	Rabbit	1:200	234167, Sigma Aldrich
Eda-Fn (Anti-Fibronectin (EDA))	Mouse	1:200	AG-20B-6001PF-C100 AdipoGen
Secondary antibodies			
Donkey anti-Mouse IgG, Alexa Fluor 488	Donkey	1:1000	A-21202, Invitrogen
Donkey anti-Rabbit IgG, Alexa Fluor 488	Donkey	1:1000	A-21206, Invitrogen

**Table 2:** Reverse transcription-quantitative PCR primer sequences.

Gene	Forward Primer 5′ → 3′	Reverse Primer 5′ → 3′
*αSma*	ACCTTCAATGTCCCTGCCATGTA	ACGAAGGAATAGCCACGCTCA
*ßAct*	GGACCTGACAGACTACCTCA	GTTGCCAATAGTGATGACCT
*Col1a1*	CCCTAATGGTGAGACGTGGA	CTTGGGTCCCTCGACTCCTA
*Col1a2*	CAGCTCCACTCTCACCTG	CAAGCCGGGAGAAAGGG
*Ctgf*	TGACCTGGAGGAAAACATTAAGA	AGCCCTGTATGTCTTCACACTG
*Eda-fn*	GGCAAGTTTCCAGGTACAGG	GCAAGGCAACCACACTGACT
*Gapdh*	AGACAGCCGCATCTTCTTGT	CTTGCCGTGGGTAGAGTCAT
*Igf*	ATCCGGCGAGGCAATAACAT	ACGGATGTGGTCGTTTTCCA
*Il-6*	CCGGAGAGGAGACTTCACAG	ACAGTGCATCATCGCTGTTC
*Lox*	GGCACCGACCTGGATATGGCACC	CGGTGAAATGGTGCAGCCTGAGG
*Lox1*	CGTCGTTACTCGGCATAGCCT	CCATGCTGTGGTAATGTTGGTG
*Mmp2*	ATCTGGTGTCTCCCTTACGG	GTGCAGTGATGTCCGACAAC
*Mmp9*	CTTCGAGGGCCACTCCTACT	CAGTGACGTCGGCTCGAGT
*Nppa*	CAGGGCCTCAAGGCACTTTT	GGTGGTCTAGCAGGTTCTTGAAA
*Nppb*	CAGCTCTCAAAGGACCAAGG	CGGTCTATCTTCTGCCCAAA
*Opn*	GAAGCCAGCCAAGGTAAGC	CACTGCCAGTCTCATGGTTG
*Postn*	ACACGGCATGGTTATTCCTTC	GAAGTCTTGGATGGAGGTGC
*Pdgfr-α*	AGGCTTGGGGCTCACTTTTT	CTCGGCCCTGTGAGGAGA
*Sm22*	AAGCAGATGGAACAGGTGGC	TGCCCAAAGCCATTACAGTCC
*SMemb*	GTGAAGCCTCTCCTCCAAGTG	TCCGTTCCATCTCCTCAAGTTC
*Tgf-ß*	AACATGCACACCCCCAAGAT	CAGGGCCTCAAGGCACTTTT
*Timp1*	ACGCTAGAGCAGATACCACG	AGCGTCGAATCCTTTGAGCA
*Thy1*	TCCTGCTTTCAGTCTTGCAG	TCATGCTGGATGGGCAAGTT
*Vegf*	GCCCTGAGTCAAGAGGACAG	CAGGCTCCTGATTCTTCCAG

## Data Availability

All data supporting the conclusions of this article are included within the article in the main text or the Supplementary Materials. RNA sequencing data sets are available via the NCBI Gene Expression Omnibus GSE238154.

## Supplementary Materials

The following supporting information can be downloaded at: https://www.mdpi.com/article/10.3390/ijms25063268/s1.
