# Supplementary material for: Microcurrent-Mediated Modulation of Myofibroblasts for Cardiac Repair and Regeneration
Source: Int J Mol Sci. 2024 Mar 13;25(6):3268. doi: 10.3390/ijms25063268 (PMC10970173; doi:10.3390/ijms25063268)
Supplement: Supplementary file 1 [file ijms-25-03268-s001.zip › MS_link access RNAseq data GEO.pdf]

GEO accession number: GSE238154

Reviewer link: https://www.ncbi.nlm.nih.gov/geo/query/acc.cgi?acc=GSE238154

Reviewer token: czsncyiufrodtqt
